# Biopolymeric Coatings for Local Release of Therapeutics from Biomedical Implants

**DOI:** 10.1002/advs.202207603

**Published:** 2023-02-13

**Authors:** Sepehr Talebian, Bárbara Mendes, João Conniot, Syamak Farajikhah, Fariba Dehghani, Zhongyan Li, Diogo Bitoque, Gabriela Silva, Sina Naficy, João Conde, Gordon G. Wallace

**Affiliations:** ^1^ School of Chemical and Biomolecular Engineering The University of Sydney Sydney NSW 2006 Australia; ^2^ Nano Institute (Sydney Nano) The University of Sydney Sydney NSW 2006 Australia; ^3^ ToxOmics NOVA Medical School|Faculdade de Ciências Médicas NMS|FCM Universidade Nova de Lisboa Lisboa 1169‐056 Portugal; ^4^ Intelligent Polymer Research Institute ARC Centre of Excellence for Electromaterials Science AIIM Facility University of Wollongong Sydney NSW 2522 Australia

**Keywords:** biopolymers, drug delivery, medical Implants, controlled release

## Abstract

The deployment of structures that enable localized release of bioactive molecules can result in more efficacious treatment of disease and better integration of implantable bionic devices. The strategic design of a biopolymeric coating can be used to engineer the optimal release profile depending on the task at hand. As illustrative examples, here advances in delivery of drugs from bone, brain, ocular, and cardiovascular implants are reviewed. These areas are focused to highlight that both hard and soft tissue implants can benefit from controlled localized delivery. The composition of biopolymers used to achieve appropriate delivery to the selected tissue types, and their corresponding outcomes are brought to the fore. To conclude, key factors in designing drug‐loaded biopolymeric coatings for biomedical implants are highlighted.

## Graphical abstract

Strategic design of a biopolymeric coating can be used to engineer the optimal release profile of the therapeutics depending on the task at hand. Here advances in delivery of therapeutics from bone, brain, ocular, and cardiovascular implants are reviewed.

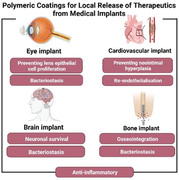

## 1 Introduction

Loss of tissue function due to degenerative diseases and trauma compromises quality of life. This is most critical in cases where the inflicted damage is beyond the natural healing ability of the tissue, requiring biomedical implant insertion to extend the functional lifetime of the injured tissue.^[^ ^1^ ^]^ These implants have been designed to fulfil one of the following tasks: either to provide physical support to hard tissues such as with bone implants,^[^ ^2^ ^]^ to replicate the movement function of a healthy joint such as with hip and knee implants,^[^ ^3^ ^]^ to impart support for vessels and tubular organs such as with coronary artery stents,^[^ ^4^, ^5^ ^]^ or, lastly, to enhance the functionality of soft tissue organs such as in the case of brain and ocular implants.^[^ ^6^ ^]^ However, after insertion, biomedical implants impose a set of ensuing problems which can jeopardize their performance. For instance, peri‐implant infections associated with orthopedic operations has been reported to be as high as 56% among patients.^[^ ^7^, ^8^ ^]^ These procedures can also induce acute inflammatory responses that can eventually lead to fibrosis or fibrous encapsulation around them, which compromises the efficiency of the device and leads to device failure.^[^ ^9^ ^]^ Similarly, loosening of joint prosthetics has been associated with macrophage responses to implant debris particles, inducing release of proinflammatory cytokines that stimulates osteoclastogenesis and ensuing excessive bone resorption.^[^ ^10^ ^]^ Glial scar formation and low neuronal density in the vicinity of chronically implanted neural electrodes have also been correlated with implant‐induced inflammatory response.^[^ ^11^ ^]^

**Figure 1:**
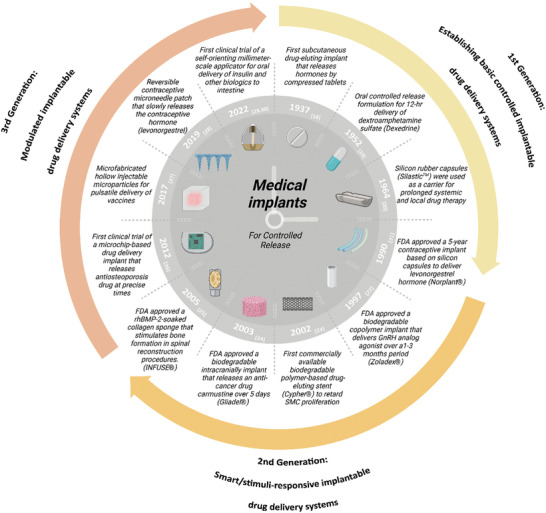
Timeline of medical implants introduced for the controlled‐release of therapeutic agents.^[^ ^18^, ^19^, ^20^, ^21^, ^22^, ^23^, ^24^, ^25^, ^26^, ^27^, ^28^, ^29^, ^30^ ^]^ FDA, Food and Drug Administration. GnRH, gonadotropin‐releasing hormone. SMC, smooth muscle cell. rhBMP‐2, recombinant human bone morphogenetic protein‐2.

As a consequence, local release of therapeutics from medical implants has been used in preclinical and clinical studies in order to circumvent infection or ameliorate acute inflammatory responses,^[^ ^12^, ^13^, ^14^ ^]^ encourage tissue integration and prolong implant life‐time. The local release approach also addresses limitations associated with systemic administration of therapeutics (e.g., low targeting efficiency and possible toxicity to nontarget tissues).^[^ ^15^, ^16^ ^]^ Such promising observations have propelled the medical device community to merge various therapeutics with implantable devices.^[^ ^17^ ^]^ From the development of the first subcutaneous drug‐eluting implant in the 1930s, biomedical implants have had tremendous success in prolonging drug administration in a precise and efficient manner (**Figure** **1**).

**Figure 2:**
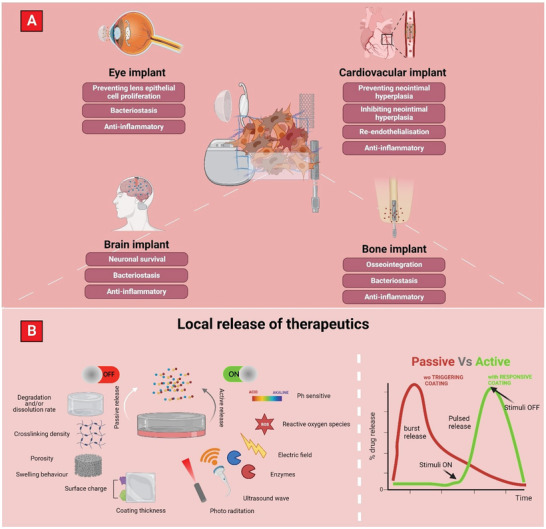
A) Graphic depicting local therapeutic release from both hard‐ and soft‐tissue implants and their associated benefits. B) Different mechanisms used to facilitate therapeutic release from bone, brain, ocular, and cardiovascular implants. Therapeutic release profile from nonresponsive and responsive biopolymer coatings.

Traditionally, implants were coated/diffused with neat therapeutics which resulted in shortcomings such as lack of control over release, loss of bioactivity, limited bioavailability, and nonuniform tissue distribution.^[^ ^31^ ^]^ To amend these issues, controlled release of therapeutics from medical implants was achieved via deployment of biopolymeric coatings (**Figure** **2**A). Biopolymers offer favorable properties (such as biocompatibility, biodegradability, and ease of processing), with their composition and structure determining the release profiles attainable.^[^ ^32^, ^33^, ^34^, ^35^ ^]^ Furthermore, crosslinked networks of polymers can yield a wide range of stiffness (from 0.5 KPa to 50 MPa), allowing them to mimic the physical properties of different tissues in the human body.^[^ ^36^ ^]^ Control over the crosslinking density of the polymeric networks can also be used to fine tune the release profile of embedded therapeutics. Polymeric coatings are typically applied to the surface of the implants using techniques including but not limited to dip‐coating, electrospinning, layer‐by‐layer self‐assembly, and surface grafting, with/without catechol mediating layer, and electrochemical deposition.^[^ ^37^, ^38^, ^39^, ^40^ ^]^ Release of therapeutics from polymeric coatings can occur passively by diffusion, or actively in response to environmental triggers (such as pH change, or certain enzymes) or external stimuli (such as ultrasound wave, photoradiation, or electric fields) (Figure 2B). Given the importance of the temporal release profile on the therapeutic effect,^[^ ^41^ ^]^ active release mechanisms attainable by smart biopolymers have gained ground to facilitate on‐demand release of therapeutics.

Responsive biopolymers can sense specific signals in the operational environment or biological processes to then trigger release of therapeutics. Responsive release modalities provide a unique opportunity to time the therapeutic administration commensurate with the biological events that ensue implant insertion. This is particularly important in providing short‐ and long‐term protection against peri‐implant infection by using particular trigger events that are ensuing the infection initiation.^[^ ^42^ ^]^

Generally, controlled release of therapeutics from medical implants has been employed to bring about one or more of the following: i) to facilitate implant integration, ii) to control host tissue inflammation, and/or iii) to prevent implant associated infections.^[^ ^33^ ^]^ This paper reviews advances in delivery of therapeutics from bone, cardiovascular, ocular, and neural implants. We have focused on these areas to highlight that both hard and soft tissue implants can benefit from controlled localized delivery. We have reviewed the biopolymeric coating systems used to facilitate therapeutics delivery from these implants, with a special focus on the chemistry of biopolymers and techniques that were used to encapsulate the therapeutics. Lastly, future trends in development of drug‐eluting biomedical implants are highlighted.

## 2 Therapeutics Release from Bone Implants

Bone tissue, unlike many others in the human body, is capable of self‐healing and being structurally integrated with surrounding undamaged counterparts, without production of any fibrotic scars.^[^ ^43^ ^]^ However, in certain cases including traumatic injuries, cancer, and osteoporosis, full recovery of bone structure and function are hampered leading to debilitating effects.^[^ ^44^ ^]^ To this end, clinical methods such as autografts and allografts have been used as gold standards to treating bone diseases, yet these approaches are often associated with issues such as disease transmission, limited autologous resources, and rejection of allograft tissue.^[^ ^45^ ^]^ Alternatively, the use of bone implants overcomes the above issues and can also provide mechanical support allowing functional recovery.^[^ ^46^ ^]^

Bone implants can be categorized into nonload bearing and load bearing implants, and correspondingly a wide range of materials have been used to manufacture them; including metallic alloys (such as titanium or magnesium), ceramics (such as hydroxyapatite or calcium phosphates), and polymers (polyether ether ketone, PEEK).^[^ ^46^ ^]^

**Figure 3:**
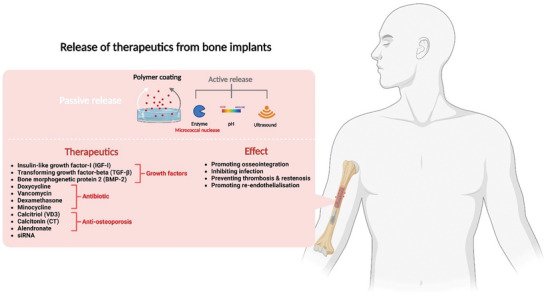
Graphic depicting the mechanism used to facilitate therapeutic release from bone implants, as well as a list of therapeutics used in these implants and their associated effects.

Titanium and its alloys are low density materials that possess high strength and resistance to corrosion, and on account of such properties they have been used frequently to manufacture medical implants.^[^ ^47^ ^]^ Titanium‐based bone implants have been widely used in clinical applications either as permanent prostheses, or as temporary implants. Nevertheless, titanium implants are prone to issues such as bacterial infection and weak osseointegration that could jeopardize their long‐term clinical application.^[^ ^48^, ^49^, ^50^ ^]^ To address these, therapeutic‐eluting bone implants have been developed (**Figure** **3**).^[^ ^51^, ^52^ ^]^ Release of therapeutics from these implants is instigated via application of a drug‐loaded biopolymeric coating.^[^ ^33^ ^]^ The nature of the coating polymer can dictate the coating modality, and sometimes mediator layers (such as polydopamine) are required to enhance the adhesion of the coating to the substrate.^[^ ^53^, ^54^, ^55^ ^]^

In the polymeric coating systems, the therapeutics are either: i) physically entrapped within the polymer matrix, or ii) they are anchored onto the polymeric chains via chemical linkages.

The release of therapeutics that are physically entrapped within the polymer coating is often passive, and the release rate is determined by polymer properties such as surface charge,^[^ ^56^, ^57^ ^]^ polymer‐drug affinity,^[^ ^58^ ^]^ concentration,^[^ ^59^ ^]^ crosslinking density,^[^ ^60^ ^]^ degradation rate,^[^ ^61^ ^]^ and swelling behavior.^[^ ^62^, ^63^ ^]^ These systems have been used to endow release of therapeutics from bone implants, mainly due to their simplicity and easy chemistries.

**Figure 4:**
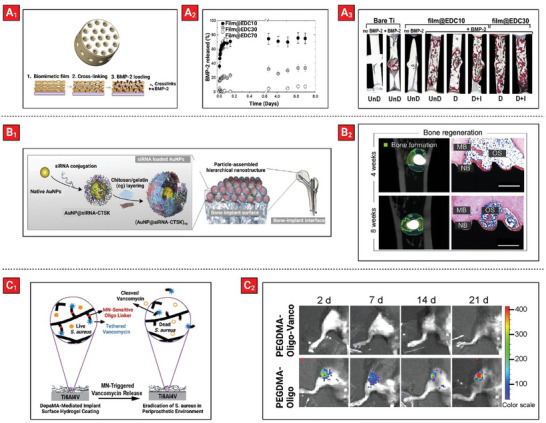
Examples of therapeutic‐loaded polymeric coatings on titanium bone implants including: A_1_) Polyelectrolyte multilayers composed of poly‐l‐lysine and hyaluronic acid crosslinked with EDC coated on porous titanium implant for BMP‐2 delivery. A_2_) Release profiles of BMP‐2 from every class of PEM film (containing different amounts of EDC) conducted in a Hepes–NaCl buffer versus time over a period of 7 days. A_3_) Cross‐sectional histological analysis of one pore channel for every class of scaffold tested for 6 weeks after implantation. Scaffolds were either undried (UnD), dried D), or both dried and *γ*‐irradiated at 25 kGy (D + I) prior to implantation. Reproduced with permission.^[^ ^60^ ^]^ Copyright 2013, Elsevier. B_1_) Polyelectrolyte multilayers composed of chitosan and gelatin loaded with Cathepsin K siRNA gold nanoparticles coated on titanium implant. B_2_) In vivo therapeutic effects of coated titanium implants in an osteoporosis rat model. NB: new bone, MB: mineralized bone, OS: osteoid. The scale bars are 50 µm. Reproduced with permission.^[^ ^71^ ^]^ Copyright 2020, Elsevier. C_1_) Poly(ethylene glycol) dimethacrylate tethered with vancomycin via an oligonucleotide linker (sensitive to *S. aureus* micrococcal nuclease; MN) coated on titanium implant. Reproduced with permission. C_2_) Total elimination of *S. aureus* inoculated in the mouse femoral canal by PEGDMA‐Oligo‐Vanco coating at 2, 7, 14, and 21 days. Reproduced with permission.^[^ ^72^ ^]^ Copyright 2019, American Chemical Society.

Titanium plates have been coated with biodegradable poly(d,l‐lactide) for local delivery of growth factors (GFs), namely insulin‐like GF‐I (IGF‐I) and transforming GF‐beta 1 (TGF‐*β*1).^[^ ^64^ ^]^ After 42 days of osteotomy, the GF‐treated rat group showed an enhanced biomechanical stability and a significantly higher callus density (mineralized area of the callus) when compared to the uncoated group. This study also emphasized the importance of the manner that GFs are delivered in‐situ, since the direct release of GFs into the medullary canal demonstrated a higher bone healing than the extramedullary GFs release. This homopolymer does not allow tuning of the therapeutic release profile, however block copolymers containing multiple repeating units can be implemented to endow some level of control over passive delivery of therapeutics.^[^ ^63^ ^]^ As an alternative approach, the LbL self‐assembly technique allows a homogenous surface coating of implants with control over thickness.^[^ ^65^, ^66^ ^]^ For example, a novel drug release system was developed on the basis of LbL deposition of poly(acrylic acid) (PAA) and poly‐l‐lysine (PLL) coatings on titanium.^[^ ^67^ ^]^ Titanium discs were first plasma etched in argon, and subsequently subjected to alternating PAA and PLL solutions. Penetration of an antibiotic (tetracycline; TC) into the coating was achieved by placing droplets of TC solution on top of the coating at 37 °C for 4 days. In vitro results showed that the implants were capable of releasing TC over a period of 15 days in both neutral (pH = 7.4) and acidic (pH = 4.5) conditions. The pH‐dependent 2‐fold increase in TC release from titanium implants observed at pH = 4.5 was attributed to changes in the degree of ionization of the polyelectrolytes, which completely eradicates *Porphyromonas gingivalis* bacterial colonies in vitro. LbL self‐assembled coatings provide a platform for pH‐sensitive release of therapeutics, mainly due to the interaction of oppositely charged polymers that form ionically crosslinked polyelectrolyte coating complexes.^[^ ^68^ ^]^ To further reinforce this network structure, crosslinking agents can be incorporated into the polyelectrolyte complex coatings.^[^ ^69^, ^70^ ^]^ For example, Ti–6Al–4 V alloy scaffolds were subjected to LbL assembly using PLL as a polycation and hyaluronic acid as a polyanion.^[^ ^60^ ^]^ These polyelectrolyte multilayer coatings were then crosslinked using different amounts of 1‐ethyl‐3‐(3‐dimethylaminopropyl)carbodiimide (EDC), and subsequently incubated with bone morphogenetic protein 2 (BMP‐2) (**Figure** **4**A_1_). The initial burst release of BMP‐2 was inhibited by increasing the EDC crosslinker concentration in the multilayer films (Figure 4A_2_). A rat ectopic model showed that the use of the crosslinked coatings resulted in better osteoinductive performance than scaffolds containing adsorbed BMP‐2 (Figure 4A_3_).

The burst release of therapeutics from polyelectrolyte complexes can be reduced by the incorporation of molecules that have an affinity toward the bioactive ingredient.^[^ ^73^ ^]^ For instance, *β*‐cyclodextrin has been grafted onto a chitosan backbone (Chi‐CD) and coated on Ti6Al7Nb substrate via LbL self‐assembly to control the release of 1,25‐dihydroxy‐vitamin D3 (VD3) and calcitonin (antiosteoporosis drug; CT).^[^ ^58^ ^]^ The coated implants showed a sustained release of therapeutic agents over a period of 14 days, which upregulates the expression of calcium‐binding proteins and BMP2 and then stimulates in vitro calcium deposition and cell differentiation. Given the essential role of macrophages in repairing bone tissue, the response of RAW264.7 cells (a murine macrophage cell line) to coated implants was evaluated, which showed that these implants could suppress the secretion of osteoclastogenesis‐related cytokines. The in vivo testing of VD3/CT coloaded implant in osteoporotic conditions showed remarkable enhanced osseointegration and the highest interfacial binding strength. Another approach used to modify the burst release of therapeutics is the incorporation of the therapeutical agents in nanoparticles and their further loading into the polyelectrolytes complexes.^[^ ^74^, ^75^ ^]^ For example, gold nanoparticles were decorated with siRNA molecules targeting Cathepsin K (CTSK; a protease involved in osteoclasts differentiation, and a regulator of vascularization) and then assembled onto a titanium implant by LbL deposition of chitosan and gelatin polyelectrolytes (Figure 4B_1_).^[^ ^71^ ^]^ This system enabled sustained release of siRNA nanovectors over a period of 8 days when tested in vitro. When cultured with RAW264.7 cells, they reduced the expression of CTSK mRNA and caused macrophage‐induced synergy in up‐regulation of at least seven bone and vascular growth factors. Insertion of these implants in an osteoporosis rat model significantly enhanced osseointegration effects (Figure 4B_2_) with improved angiogenesis of blood vessels associated with the regenerated bone.

The therapeutics agents can also be anchored onto the polymer coating through chemical linkers that can anchor therapeutic agents on the implant surface. Depending on the type of chemical linkage between the therapeutic and the polymer, either permanent immobilization^[^ ^76^, ^77^ ^]^ or stimuli‐responsive release can be achieved.^[^ ^72^ ^]^ The immobilization of therapeutics can be a useful approach in cases where long‐term therapeutic effects are expected from the implant. On the other hand, stimuli‐responsive release is ideal in scenarios where drugs need to be delivered at specific time points and follow a certain pattern of release to fulfil their optimal therapeutic effect.^[^ ^78^ ^]^ Permanent immobilization of therapeutics onto the polymer coatings are achieved using chemistries such as Michael addition,^[^ ^79^, ^80^ ^]^ Schiff base reaction,^[^ ^76^, ^81^, ^82^ ^]^ epoxide ring‐opening reaction,^[^ ^83^ ^]^ click chemistry,^[^ ^84^ ^]^ and radical initiated reaction.^[^ ^85^ ^]^

Ethanediamine‐functionalized poly(glycidyl methacrylate) (PGED) brushes have been grafted onto titanium implants using surface‐initiated atom transfer radical polymerization.^[^ ^76^ ^]^ Then, the polymer brushes have been further functionalized with low‐molecular‐weight quaternized polyethyleneimine and alendronate, an antibacterial and antiosteoporosis agent, respectively. Biomedical device‐associated infections animal model indicated that implants inhibited bacterial infection and provided a supportive environment for bone‐implant osseointegration leading to high biomechanical stability of implants. In other work, a titanium implant was coated with polydopamine to facilitate conjugation of the antibiotic cefotaxime sodium (CS) onto the surface of the implant.^[^ ^79^ ^]^ The immobilization of drug on the polydopamine coating was achieved via Michael addition and Schiff‐base reactions between the amino groups in CS and the catechol/quinone groups in polydopamine. In vitro results showed that the antibiotic‐grafted titanium effectively prevented adhesion and proliferation of two different bacteria. Despite the success of permanently immobilized therapeutics on the polymer coating, the therapeutic effect of these systems is restricted to the immediate surface of the implant. Consequently, stimuli‐responsive chemical linkages such as peptide bonds that are sensitive to bacterial enzymatic activity have emerged as a useful strategy to induce the release of therapeutics upon infection.^[^ ^72^, ^86^ ^]^ Vancomycin (a glycopeptide antibiotic) has been covalently attached to poly(ethylene glycol) dimethacrylate hydrogel coatings using a nuclease sensitive oligonucleotide linker, to endow sensitivity to *S. aureus* micrococcal nuclease (Figure 4C_1_).^[^ ^72^ ^]^ The insertion of these coated titanium pins into mouse femoral canal inoculated with *S. aureus* resulted in complete eradication of the bacteria from both the implant surface and its periprosthetic bony tissue environment, preventing osteolysis in the surrounding tissue (Figure 4C_2_).

Therapeutic‐loaded polymeric coatings provide a unique opportunity to provide control over the release profile, due to the diverse chemistries that can be engineered to endow specific spatiotemporal release patterns. However, factors such as polymer degradation products could potentially induce negative immune‐system responses, which could be detrimental to the performance of the implant. For instance, polylactic acid degradation generates acidic products (lactic and glycolic acids) that lowers the pH, and this, in turn, negatively affects the cytokine profiles of inflammatory cells adjacent to the implants.^[^ ^87^ ^]^ Also, the polymer degradation rate must be adjusted carefully to allow attachment of newly formed bone to the surface of the implant, ensuring complete osseointegration.^[^ ^88^ ^]^ Application of functional nanoparticle‐loaded polymeric coatings on bone implants has recently gained a lot of attention. Owing to peculiar properties of these nanoparticles they can either be used for delivery of specific genes to facilitate osseointegration, or they can endow photothermal and photodynamic properties to the implants to prevent periimplant infections.^[^ ^71^, ^89^, ^90^, ^91^ ^]^ A summary of therapeutic‐releasing biopolymeric coatings on bone implants is provided in **Table** **1**.

**Table 1:** Summary of therapeutic‐eluting bone implants

Type of implant	Type of biopolymer(s) coating	Therapeutic Type	Mode of delivery	In vivo model	Results	Ref.
Bone (titanium)	Poly(d,l‐lactide)	Growth factor (IGF‐I & TGF‐*β*1)	Passive	Osteotomy in rats	After 42 days significant improvements were observed in callus density and maximum load density	[64]
Bone (PEEK)	Polydopamine	Antibiotic (dexamethasone and minocycline loaded in liposomes)	Passive	Subcutaneous implant‐associated infection in mice, and femur of beagle dogs	Improved the local anti‐inflammatory and bacteriostasis effect in subcutaneous model. Promoted a larger area of new bone formation in dogs	[77]
Bone (titanium)	Core–shell nanofibers containing polyvinyl alcohol (core) and polycaprolactone (shell)	Antibiotic (doxycycline)	Passive	Infected tibia implantation rat model	Inhibition of bacterial growth up to 8 weeks, and enhanced osseointegration	[92]
Bone (titanium)	Multilayer polyelectrolytes containing poly‐l‐lysine and hyaluronic acid crosslinked with EDC	Growth factor (BMP‐2)	Passive	Rat ectopic model	Significant osteoinductive performance when compared to that of implants with BMP‐2 physically adsorbed on their surface	[60]
Bone (PEEK)	Multilayer polyelectrolytes containing poly‐l‐lysine and hyaluronic acid	Growth factor (BMP‐2)	Passive	Femoral condyles in rabbits	High dosage of BMP‐2 release led to significantly lower bone‐to‐implant contact and bone area around the implants compared to that in bare implants.	[70]
Bone (titanium)	Multilayer polyelectrolytes containing *β*‐cyclodextrin grafted chitosan and gelatin	Antiosteoporosis drug (CT & VD3)	Passive	Osteoporotic rabbit model	After 90 days significant improvement was observed in osseointegration compared to uncoated samples	[58]
Bone (titanium)	Multilayer polyelectrolytes containing chitosan and gelatin	Gene therapy (siRNA for silencing Cathepsin K decorated on gold nanoparticles)	Passive	Osteoporotic rat model	After 56 days significantly enhanced osseointegration effects with improved angiogenesis of blood vessels associated with the regenerated bone	[71]
Bone (titanium)	Poly(glycidyl methacrylate) functionalized with the drugs	Antiosteoporosis drug (alendronate) together with antibacterial agent (quaternized polyethyleneimine)	Passive	Infected distal femoral metaphysis defect in rat	Implants inhibited bacterial infection and provided a supportive environment for bone–implant osseointegration	[76]
Bone (titanium)	Poly(ethylene glycol) dimethacrylate functionalized with the drug	Antibiotic (vancomycin)	Active‐ in response to *S. aureus* nuclease	Infected femoral canal in mice	Complete eradication of the bacteria from both the implant surface and its periprosthetic bony tissue environment. Prevention of osteolysis in the surrounding bone tissue	[72]
Bone (titanium nanotubes)	Multilayer polyelectrolytes containing chitosan–catechol and hyaluronate–catechol	Antibiotic (vancomycin)	Active‐in response to hyaluronidase enzyme	Infected femoral defect in rat	After 1‐month Improved osseointegration and prevented bacterial infection, compared to bare implants	[93]
Bone (PEEK containing macroholes)	Polylactic acid	Antibiotic (vancomycin)	Active‐in response to ultrasound wave	Ex vivo‐infected spine of cadaveric rabbit	Significantly more drug was released once the samples were exposed to ultrasound waves, which led to great inhibition of *S. aureus* growth when compared to that in uninsonated implant	[94]

## 3 Therapeutic Release from Brain Implants

Brain is the most complex tissue in the human body, and it is responsible for almost every physiological action in the body.^[^ ^95^ ^]^ Consequently, neurological complications caused by injury or disease is detrimental to an individual's quality of life by compromising their normal daily activity.^[^ ^96^ ^]^ Such neurological complications could lead to loss of hearing, vision, compromised memory, and paralysis.^[^ ^97^ ^]^ These neurological complications are associated with loss of neural function, which is worsened given that neurons are usually incapable of regeneration.^[^ ^98^ ^]^ Normally, some of the neural pathways upstream of the defective site remain functionally intact, and they can be explored via neural implants to restore lost function.^[^ ^99^ ^]^ For example, implantable‐device technologies capable of transferring electrical and chemical signals to and from the nervous system have generated possibilities to ameliorate dysfunction resulting from disease or injury.^[^ ^100^ ^]^ Such implantable devices include auditory brainstem implants; deep brain stimulators; and intracortical microelectrodes.^[^ ^101^ ^]^ However, insertion of these devices in brain tissue is often associated with local injury and a progressive inflammatory tissue response, which can interfere with electrophysiological signals, compromising the device function.^[^ ^102^ ^]^

**Figure 5:**
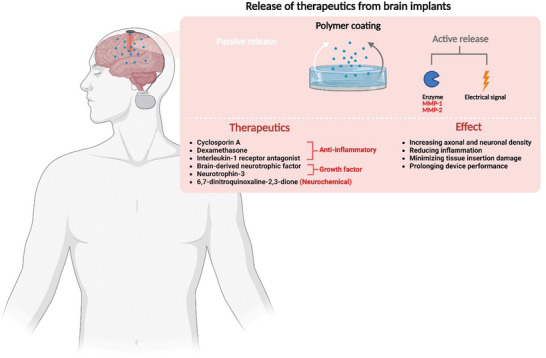
Graphic depicting the mechanism used to facilitate therapeutic release from brain implants, as well as a list of therapeutics used in these implants and their associated effects.

To address this hurdle, controlled delivery of therapeutics from neurobionic devices has been shown to provide promising results (**Figure** **5**).^[^ ^103^, ^104^, ^105^, ^106^, ^107^ ^]^ These devices normally contain electrode arrays that are insulated with soft and flexible polymeric materials to make them more compatible with the curved and easily‐damaged surface of the brain.^[^ ^105^ ^]^ Polydimethylsiloxanes (PDMS) and polyimides (PI) are two of the most prevalently used insulating polymers, mainly due to their favorable features such as flexibility, chemical and radiation inertness, durability, and good biocompatibility.^[^ ^108^, ^109^ ^]^

The local release of neurotrophins and/or anti‐inflammatory drugs from these devices is achieved either through release from the insulating polymer^[^ ^110^, ^111^ ^]^ or via polymeric coatings on top of these. The insulating polymer can only afford passive release of therapeutics without any control over the release rate.^[^ ^112^ ^]^ On the other hand, polymer coating can provide a degree of versatility to tune the release profile either in a passive or active manner.

**Figure 6:**
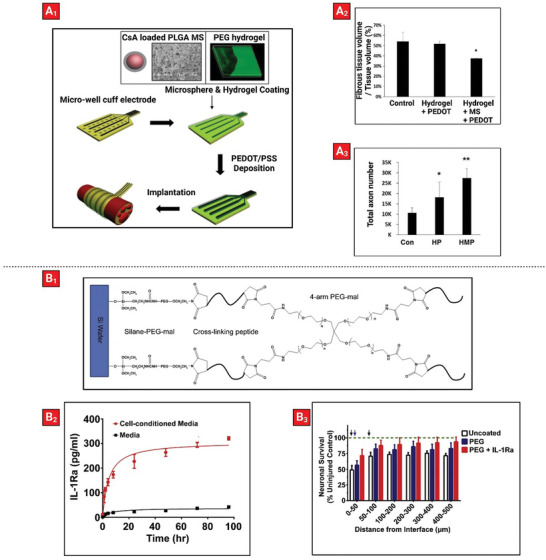
Example of therapeutic‐loaded polymeric coatings on brain implant. A_1_) Nerve cuff electrode coated with polyethylene glycol (PEG) hydrogel encapsulated with poly(lactic‐*co*‐glycolic) acid (PLGA) microspheres (MS) containing cyclosporin A (CsA) and topped off with a conductive polymer (PEDOT/PSS). A_2_) Fibrous tissue deposits after 5 weeks implantation with different surface coated electrodes. A_3_) Sciatic nerves analysis of implanted coated electrodes after 5 weeks of insertion. Reproduced with permission.^[^ ^114^ ^]^ Copyright 2016, Elsevier. B_1_) Silicon substrate of a neural electrode coated with polyethylene glycol hydrogel containing interleukin‐1 receptor antagonist (IL‐1Ra) and crosslinked with a protease‐sensitive peptide. B_2_) IL‐1Ra release profiles exhibiting a faster release with LPS‐stimulated cell media compared to media alone. B_3_) Neuronal survival around the electrode. Green dotted line showing uninjured control. Reproduced with permission.^[^ ^115^ ^]^ Copyright 2015, Elsevier.

In brain/neural implants, biopolymer coatings are the sole candidate for drug delivery, mainly due to the soft and delicate nature of the tissue that necessitates the use of soft materials to avoid physical damage. Specifically, given the essential role of electrical signals in the performance of brain implants, electrically conducting polymer coatings have emerged as an alternative to facilitate drug release. For example, polyimide‐based nerve cuff electrode was coated with poly‐l‐lactic acid (PLLA) or poly(lactide‐*co*‐glycolic acid) (PLGA) nanofibers loaded with dexamethasone.^[^ ^113^ ^]^ Next, PEG was patterned on the nanofibers as a drug release facilitator, on top of which a conductive layer of poly(3,4‐ethylenedioxythiophene)‐poly(styrenesulfonate) (PEDOT:PSS) was polymerized to counter the loss of electrical properties caused by PEG patterning. In vitro drug release experiments showed that implants coated with PLGA nanofibers had a faster release compared to that from PLLA fibers (due to the difference in their degree of hydrophilicity), and the addition of PEG further increased the release rate. Moreover, electrodes coated with PEDOT:PSS had a lower impedance (342 Ω mm^2^) when compared to PEG‐patterned electrodes (1046 Ω mm^2^). In addition, testing of these nerve cuff electrodes in an ex vivo SD rat's sciatic nerve showed successful recording of the nerve's signals. However, in vivo evidence was lacking from this work to truly evaluate the effect of the released drug. Consequently, the same group coated a nerve cuff electrode with a PEG hydrogel embedded with PLGA microspheres (MS) containing cyclosporin A (CsA; anti‐inflammatory drug) (**Figure** **6**A_1_). Subsequently PEDOT:PSS was deposited on the four‐line electrodes site on the hybrid hydrogel‐coated cuff electrode to improve the electrode‐nerve tissue interface.^[^ ^114^ ^]^ Accordingly, these electrodes allowed controlled release of cyclosporin A over a period of 40 days when tested in vitro. Also, it was shown that PEDOT deposition can reduce the impedance of platinum electrodes. In vivo testing of the PEDOT:PSS coated electrode with PEG hydrogel and cyclosporin A containing microspheres significantly reduced fibrous tissue deposition (Figure 6A_2_) and increased axonal density (Figure 6A_3_). Moreover, histological analysis of sciatic nerves treated with coated implants containing cyclosporin A revealed a more highly packed nerve fiber as well as myelin sheath in comparison with that in control and hydrogel + PEDOT treated groups.

The cases that were discussed so far allowed sustained release of drugs from brain implants in a passive manner, yet active delivery in response to both internal^[^ ^115^ ^]^ and external stimuli^[^ ^116^, ^117^ ^]^ provides a further dimension. To avoid the electrode insertion trauma (EIT), silicon‐based electrode arrays were dip‐coated with a PEG‐maleimide hydrogel that contained an Interleukin‐1 receptor antagonist (IL‐1Ra; anti‐inflammatory drug) and subsequently crosslinked using a peptide linker (Figure 6B_1_).^[^ ^115^ ^]^ In fact, the peptide crosslinkers were protease‐sensitive, which allowed the release of IL‐1Ra in response to upregulated proteases during inflammation. First, hydrogels were placed in conditioned media from cells stimulated with lipopolysaccharide (LPS) (to induce secretion of inflammatory proteases) or normal media. Faster release of IL‐1Ra was observed overtime under simulated cell culture conditions (Figure 6B_2_). Subsequently, in vitro testing showed that these implants significantly reduced attachment of glial cells and attenuated inflammatory cytokine release, while facilitating increased release of IL‐1Ra over time in response to inflammatory cytokines. However, in vivo experiments only showed minor improvements in inflammatory cell markers, albeit neuronal survival was significantly higher for coated electrodes (Figure 6B_3_).

Electrically conductive polymers present a useful coating option for brain implants, as they facilitate large drug loading capacity as well as electrical stimulation.^[^ ^118^ ^]^ To this end, electrically conducting polypyrrole/*para*‐toluene sulfonate (PPy/pTS) containing neurotrophin‐3 (NT3; a neural growth factor) was electrochemically deposited onto cochlear implant electrodes for preventing gradual degeneration of spiral ganglion neurons (SGNs).^[^ ^116^ ^]^ The in vitro results revealed that the amount of released NT3 can be tuned by using a biphasic electrical stimulation. Of interest, implanting PPy/pTS/NT3 electrode arrays in the cochleae of guinea pigs followed by electrical simulation caused higher SGN densities in implanted cochleae when compared to that in animals implanted with PPy/pTS‐coated electrodes.

Lastly, a mixture of poly(dimethylacrylamide‐*co*‐4‐methacryloyloxy benzophenone‐*co*‐4‐styrenesulfonate) hydrogel and PEDOT coated on an electrode, provided large loading capacity of dexamethasone and actively released the drug in response to electrical signals.^[^ ^117^ ^]^ These electrodes allowed on‐demand delivery of dexamethasone over a period of 12 weeks in fully awake animals using a cyclic potential waveform. Drug‐functionalized probes showed uninterrupted performance over the entire chronic study. Of importance, histological evaluation of the tissue surrounding the probe showed that electrodes exposed to on‐demand drug release had neurons closer to the electrode sites compared to controls. A summary of therapeutic‐releasing biopolymeric coatings on brain implants is provided in **Table** **2**.

**Table 2:** Summary of therapeutic‐eluting brain implants

Type of implant	Type of biopolymer(s) coating	Therapeutic type	Mode of delivery	In vivo model	Results	Ref.
Brain (PI‐coated nerve cuff electrode)	Poly(ethylene glycol)	Anti‐inflammatory drug (cyclosporin A loaded in PLGA microspheres)	Passive	Sciatic nerve in rats	Relative fibrous tissue volume was significantly reduced, which led to increased axonal density around the implant compared to bare implants	[114]
Brain (PI‐coated microwire electrode)	Poly(ethylene glycol) dimethacrylate	Anti‐inflammatory drug (dexamethasone)	Passive	Rats cortex	Caused an overall reduction in expression of pro‐inflammatory markers at the local tissue	[119]
Brain (PI‐coated cochlear implant electrode)	Alginate functionalized with arginine‐glycine‐aspartic acid (RGD)	Brain‐derived neurotrophic factor (BDNF)	Passive	Cochleae of guinea pigs	Released a significant ratio of bioactive BDNF into the cochlear fluids (28.14 ng mL^−1^) within the first week of implantation, which was subsequently dropped to almost half of its initial value (13.91 ng mL^−1^) during the second week of experiments	[120]
Brain (PDMS‐coated neural microelectrodes)	Alginate	Anti‐inflammatory drug (dexamethasone loaded in PLGA nanoparticles)	Passive	Guinea pigs auditory cortex	After two weeks of implantation, dexamethasone loaded electrodes showed a steady level of impedance, as compared to 3 times increase for the control electrodes.	[121]
Brain (PI‐coated neural microelectrode array)	Poly(ethylene oxide)	Anti‐inflammatory drug (dexamethasone loaded in poly(propylene sulfide) nanoparticles)	Passive	Rats primary motor cortex	Prolonged reduction in the tissue response as compared to noncoated electrodes.	[122]
Brain (PI‐coated neural microelectrodes)	Poly(3,4‐ethylenedioxythiophene)	Anti‐inflammatory drug (dexamethasone)	Active‐in response to electrical signals	Rats brain	On‐demand released drug caused closer neuronal density to the electrode in comparison to control.	[117]
Brain (PDMS‐coated cochlear implant electrode)	Polypyrrole/*para*‐toluene sulfonate	Neural growth factor (neurotrophin‐3)	Active‐ in response to electrical signals	Cochleae of guinea pigs	Electrically stimulated drug‐loaded implants induced greater spiral ganglion neurons density, and did not exacerbate fibrous tissue formation	[116]
Brain (PDMS‐coated microelectrode array)	Dual layer containing poly(3,4‐ethylenedioxythiophene/acid functionalized carbon nanotube, and polypyrrole/acid functionalized carbon nanotube	Neurochemical (6,7‐dinitroquinoxaline‐2,3‐dione)	Active‐ in response to electrical signal	Rat somatosensory cortex	On‐demand release of drug caused immediate suppression of neural activity in the vicinity of the electrodes.	[123]
Brain (PDMS‐coated electrode arrays)	Poly(ethylene glycol)‐maleimide crosslinked with a peptide linker	Anti‐inflammatory drug (Interleukin‐1 receptor antagonist)	Active‐ in response to enzyme	Rats brain	Negligible enhancement in expression of inflammatory cell markers, but caused significantly higher neuronal survival for coated electrodes.	[115]

## 4 Therapeutic Release from Ocular Implants

The eye is divided into two main anatomical regions: the anterior and posterior segments. Various diseases (inflammatory, infectious, degenerative, and hereditary) can harm regions in both anterior and posterior segments of the eye.^[^ ^124^ ^]^ The gold standard for delivering drugs to the eye are the eye drops and it is responsible for 90% of all ophthalmic medications. However, using eye drops, less than 5% of the drug penetrates through the cornea. Drug absorption is hindered by static barriers such as the corneal epithelium, stroma and endothelium, blood‐aqueous barrier and dynamic barriers such as tear dilution, conjunctival barrier, and blood‐retinal barrier.^[^ ^125^ ^]^ As a result, repeated dosing (up to 4 times per day for many treatments) is necessary which has given rise to fluctuations in drug concentration over the ocular structures. To amend this, ocular lenses (contact lens, and intraocular lens) were suggested as a platform to allow local and sustained release of therapeutics to the eye. This will enhance the ocular absorption and consequently avoid the possible negative side effects of the drugs associated with multiple administrations.

Depending on the material used for preparation, ocular lenses can be classified as hard or soft. Generally hard lenses are made up of rigid polymers such as polymethyl methacrylate (PMMA), whilst soft lenses are made from soft and flexible polymers such as poly (2‐hydroxyethyl methacrylate) (pHEMA)‐based hydrogel or silicone hydrogel.^[^ ^126^ ^]^ Control of drug release from contact lenses is essential, ensuring adequate concentration in the eye to yield optimal therapeutic effect over the treatment period. Compared to drug‐soaked lenses (drug adsorbed physically on the surface), that are prone to burst release and low drug loading capacity, emerging ocular lenses made from techniques such as vitamin E barriers,^[^ ^127^, ^128^, ^129^ ^]^ molecular imprinting,^[^ ^130^, ^131^, ^132^, ^133^, ^134^ ^]^ micro‐ and nanoparticles laden,^[^ ^135^, ^136^, ^137^, ^138^, ^139^, ^140^ ^]^ inner layer‐embedded lenses^[^ ^141^, ^142^, ^143^, ^144^, ^145^, ^146^ ^]^ can facilitate the maintenance of local dosage (within a therapeutic window) for a longer period of time. Despite such achievements, premature release during manufacturing and storage have hindered the potential application of drug eluting ocular lenses in clinical use. Moreover, timely mannered drug dosage adjustment is imperative as the disease/condition is progressing. However, the common theme among drug‐eluting ocular lenses is release at a predetermined rate which is not compatible with the changing physiological conditions of the disease.

**Figure 7:**
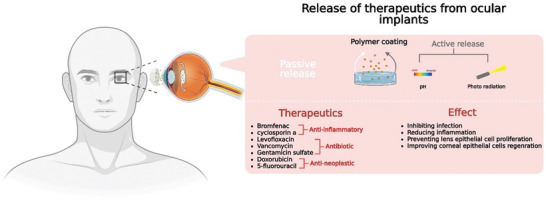
Graphic depicting the mechanism used to facilitate therapeutic release from ocular implants, as well as a list of therapeutics used in these implants and their associated effects.

Consequently, biopolymer coatings were applied onto the surface of lenses to inhibit premature drug elution and assist in providing on‐demand release of therapeutics (**Figure** **7**). The coating layer is applied onto the surface of the contact lens either via spray‐coating,^[^ ^147^ ^]^ or spin‐coating,^[^ ^148^ ^]^ or through LbL deposition^[^ ^149^, ^150^, ^151^, ^152^, ^153^ ^]^ of biopolymeric layers.

A concentric ring‐patterned cyclosporin A (CsA)‐loaded PLGA was spin‐coated onto the surface of an intraocular lens (IOL) to effectively prevent posterior capsular opacification.^[^ ^148^ ^]^ This is the main ensuing condition after IOL implantation in cataract surgery, because of residual lens epithelial cells (LECs) proliferation in the lens capsule. Given the transmittance requirements of the intraocular lens, concentric annular coating with a thin center and thick periphery was obtained. In vitro drug release profiles obtained from these systems showed a four‐phase pattern that contained an initial burst release for the first week, followed by a linear release from week 2 to 9, with an ensuing secondary burst release from week 9 to 12, and ending with a plateaued release from week 12 to 16. The initial burst release had an inverse correlation with the PLGA concentration. The use of a PLGA coating only slightly decreased light transmittance of the pristine IOLs (from 87% to 83%), showing no major impact on the optical properties of IOLs. The in vitro results showed that modified IOLs were capable of inhibiting LECs proliferation and induced cell death. After two weeks of implantation in rabbit eyes, unmodified IOL showed excessive fibrosis of residual cells in the vicinity of the implant. In contrast, no posterior capsular opacification was detected in the CsA‐loaded PLGA coating‐modified IOL‐implanted animals. The results suggested that CsA‐loaded PLGA coating on the surface of IOLs was effective in the prevention of posterior capsular opacification.

**Figure 8:**
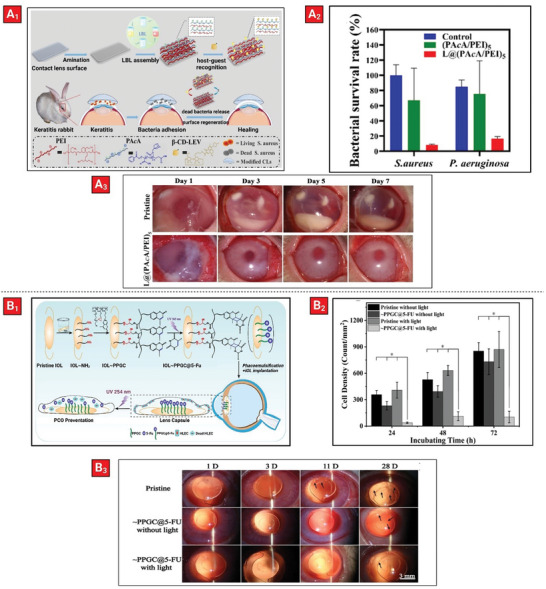
Examples of therapeutic‐loaded polymeric coatings on ocular implant. A_1_) Schematic showing the preparation of contact lens surface using layer‐by‐layer deposition and host–guest interaction. A_2_) Survival rate of bacteria adhered on different types of CLs. A_3_) Representative photographs of S. aureus infectious keratitis in rabbits’ eyes treated with different CLs after 1‐, 3‐, 5‐, and 7‐days post insertion. Reproduced with permission.^[^ ^154^ ^]^ Copyright 2022, American Chemical Society. B_1_) Schematic depicting the chemistry and photoresponsive drug release from the IOL surface coating. This is used for prevention of posterior capsular opacification after intraocular insertion. B_2_) Human lens epithelial cells (HLECs) density on various coatings in presence or absence of irradiation after incubation for 24, 48, and 72 h. B_3_) Images of postoperative eyes treated with different IOL groups at 1, 3, 11, and 28 days (black arrows showing posterior capsular opacification). Reproduced with permission.^[^ ^155^ ^]^ Copyright 2022, Elsevier.

Bacterial keratitis is a serious ophthalmic condition that can lead to severe visual impairment. In a recently published work, a combination of LBL self‐assembly and host–guest interactions was utilized to coat the surface of corneal contact lenses (CLs) with the aim of enhancing bioavailability of the drug for effective treatment of bacterial keratitis (**Figure** **8**A_1_).^[^ ^154^ ^]^ The coating layers consisted of a polyanionic copolymer of acrylic acid and 1‐adamantan‐1‐ylmethyl acrylate (P(AA‐*co*‐AdA)) and a polycationic poly(ethyleneimine) (PEI). Next, host–guest interactions between *β*‐cyclodextrin−levofloxacin (*β*‐CD‐LEV) and AdA were used to develop an antibacterial coating on the CLs. These implants were designed to be reusable by simply removing the adherent dead bacteria using sodium dodecyl sulfate (SDS) and reloading them with the drug by simply immersing them in *β*‐ CD‐LEV. By tuning the number of bilayers, one could control the drug loading capacity as well as coating thickness. In vitro (Figure 8A_2_) and in vivo (Figure 8A_3_) experiments revealed the antibacterial and germicidal efficacy of the coatings.

Recently, photo‐ and pH‐responsive coatings deposited on the surface of CLs have been used to facilitate on‐demand drug release. The photoresponsive coating on the surface of IOLs was developed based on reversible photodimerization and photolysis of coumarin and 5‐FU (Figure 8B_1_).^[^ ^155^ ^]^ The copolymer containing coumarin moiety was a poly (polyethylene glycol methacrylate‐*co*‐glycidyl methacrylate‐*co*‐Coumarin methacrylate) which was attached onto the IOL surface. 5‐FU can be attached or released from the copolymer under the action of UV radiation at 365 or 254 nm, respectively. In vitro results showed that coated IOLs succeeded in preventing LEC adhesion and proliferation via irradiation (Figure 8B_2_). The on‐demand release upon irradiation was a function of UV light intensity and radiation time. In vivo observations revealed that surface‐modified IOLs were capable of reducing the occurrence of posterior capsular opacification after light radiation with minimal side effects (Figure 8B_3_).

Along similar lines, a pH‐responsive coating was obtained via surface‐initiated reversible addition–fragmentation chain transfer polymerization (SI‐RAFT) of phenylboronic acid monomers (4‐allylaminocarbonylphenylboronic acid, ACPA) into uniform brushes.^[^ ^156^ ^]^ The dynamic covalent bonds between hydroxyls of vancomycin and phenylboronic acid (PCVB) were used to facilitate drug loading. These interactions possessed sensitivity toward acidic pH that can be generated because of bacterial infection. Concentration of the monomer and duration of SI‐RAFT polymerization determine the thickness of the coating as well as the loading capacity of the drug. Furthermore, in vitro and in vivo assessment of the PCVB coating yielded improved antibacterial and anti‐inflammatory effects. The results of this study demonstrated that the tissue environment associated with bacterial infection can be used as a trigger to allow responsive release of therapeutics, representing a potential solution for bacterial keratitis.

Drug release patterns must be consistently optimized in response to disease progression, treatment response, and other concurrently occurring conditions. In these cases, polymer coatings with active release mechanisms could be useful. Amongst external stimuli, light represents a promising trigger to initiate/control drug release, mainly because it can be easily applied remotely with high spatiotemporal precision. In this context, ultraviolet (UV) radiation can modulate the release profile of therapeutics by means of either photocleavage, photoisomerization, or photo‐crosslinking. At the same time, reloadable polymeric coatings on contact lenses provide another useful feature for longer‐term treatments. As a result, multimodal polymer coatings such as the ones with responsive release and reloadable attributes hold a great potential for improving ocular delivery of therapeutics. A summary of therapeutic‐releasing biopolymeric coatings on ocular implants is provided in **Table** **3**.

**Table 3:** Summary of therapeutic‐eluting ocular lenses

Type of implant	Type of biopolymer(s) coating	Therapeutic type	Mode of delivery	In vivo model	Results	Ref.
Intraocular lens (IOL)	PLGA	Bromfenac‐ nonsteroidal anti‐inflammatory drug	Passive	White rabbit posterior capsular opacification model (PCO)	Improved PCO prevention and suppression of inflammation, while exhibiting no toxicity.	[147]
Intraocular lens (IOL)	PLGA	Cyclosporin A (CsA)‐ immunosuppressant	Passive	Rabbit eyes through the standard cataract surgery procedure	After two weeks of implantation, no PCO was detected in animals treated with CsA‐loaded PLGA coating‐modified IOL.	[148]
Intraocular lens (IOL)	Multilayers of polydopamine and 2‐methacryloyloxyethyl phosphorylcholine (MPC)	Doxorubicin (DOX)‐ antiproliferative	Passive	White rabbit posterior capsular opacification model (PCO)	In vitro and in vivo observations showed great inhibition in both adhesion and proliferation of residual human lens epithelial cells.	[152]
Corneal contact lens	Multilayer polyelectrolytes containing polyanionic copolymer of acrylic acid and 1‐adamantan‐1‐ylmethyl acrylate (P(AA‐*co*‐AdA)) and a polycationic poly(ethyleneimine) (PEI).	*β*‐cyclodextrin–levofloxacin conjugate‐ antibiotic	Passive	S. aureus infectious keratitis induced in adult Japanese white rabbit's eye	Biocompatibility and inhibition of bacterial keratitis was observed during in vivo assessment in S. aureus keratitis models.	[154]
Intraocular lens (IOL)	Terpolymer poly (polyethylene glycol methacrylate‐*co*‐glycidyl methacrylate‐*co*‐Coumarin methacrylate) (PPGC)	5‐fluorouracil (5‐FU)‐ antiproliferative	Active‐photo responsive	White rabbit posterior capsular opacification model (PCO)	On‐demand release of 5‐FU using UV radiation with 254 nm. Intraocular implantation in animal models showed good biosafety, and the lens was capable of halting PCO progression only under 5 min of UV irradiation.	[155]
Corneal contact lens	Poly(4‐allylaminocarbonylphenylboronic acid) (PACPA)	Vancomycin (Van)‐ antibiotic	Active‐ pH responsive	Corneal *S. aureus* infection model in rats	pH‐responsive release of drug at acidic pH associated with bacterial infection. This led to significant reduction in inflammatory responses. Also, facilitated regeneration of corneal epithelial cells, with intact cornea structure.	[156]
Corneal contact lens	Multilayer of oxidized alginate and polyethylenimine (PEI)	Gentamicin sulfate (GS)‐antibiotic	Active‐ pH responsive	Corneal *S. aureus* infection model in rats	pH‐responsive release with repeatable loading capacity. Total prevention and treatment of corneal infections both in vitro and in vivo.	[157]

## 5 Therapeutic Release from Cardiovascular Stents

**Figure 9:**
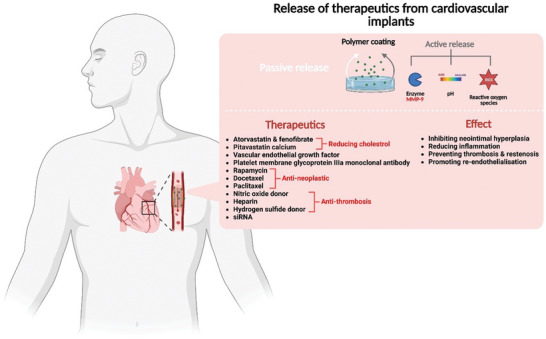
Graphic depicting the mechanism used to facilitate therapeutic release from cardiovascular implants, as well as a list of therapeutics used in these implants and their associated effects.

Cardiovascular diseases (CVDs) are one of the most prevalent chronic diseases and the leading cause of death worldwide.^[^ ^158^, ^159^ ^]^ Due to the significant morbidity and mortality due to CVDs, different medical interventions have emerged to improve treatment efficacy. Cardiovascular stents have been used as an important and versatile method to address coronary and peripheral artery diseases.^[^ ^160^, ^161^ ^]^ However, stents are bioinert metallic devices and their implantation can lead to inflammation, restenosis, and thrombosis.^[^ ^162^, ^163^ ^]^ To reduce such side‐effects, bioactive compounds such as vascular endothelial growth factor (VEGF) to support neovascularization, rapamycin and paclitaxel to suppress foreign body reactions, and heparin to prevent blood clotting, have been incorporated into stents usually with the aid of a biodegradable polymer as a coating (**Figure** **9**). Ideally, precise concentrations of the therapeutic agent should be delivered to the target position, in a timely manner and over a relatively long period of time. However, burst‐release after implantation, which leads to over‐ and under‐dosing over time, is a common issue with most drug‐loaded stents. In addition, biodegradable synthetic polymers such as PLGA and poly‐l‐lactide acid (PLLA) that are often used produce acidic degradation products, leading to local inflammation and delayed tissue healing due to the local acidification.^[^ ^163^, ^164^ ^]^ Hence, naturally derived biopolymers such as zein protein (from corn) or alginate (from seaweed), that develop less inflammation in long‐term applications, have been used to substitute synthetic biodegradable polymers.

To further modulate release of the drugs and achieve a sustained release profile, multilayer polymer coatings,^[^ ^165^, ^166^ ^]^ LbL self‐assembly,^[^ ^167^, ^168^ ^]^ and drug‐loaded nanoparticles immobilized on^[^ ^169^ ^]^/in^[^ ^170^ ^]^ the coated polymeric matrix have been utilized.

**Figure 10:**
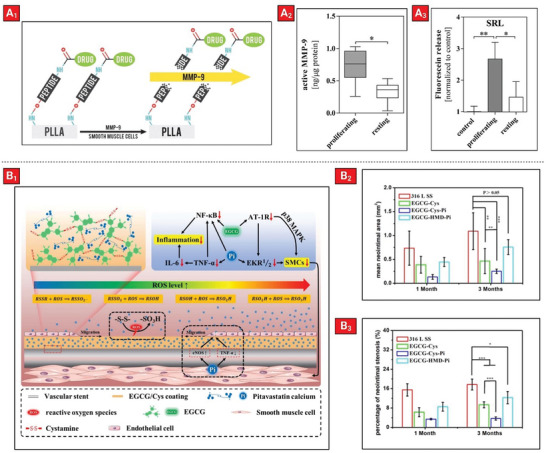
Example of therapeutic‐loaded polymeric coatings on a cardiovascular implant. A_1_) MMP‐9 cleavable peptide linkers anchored to the stent surface facilitates drug release in response to proliferation of smooth muscle cells. A_2_) Difference in the levels of active MMP‐9 between proliferating and resting HCASMC. A_3_) Release of fluorescein from polymeric coatings in response to HCASMC at proliferating or resting state. Reproduced with permission.^[^ ^171^ ^]^ Copyright 2016, American Chemical Society. B_1_) The coating was formed based on crosslinking of Epigallocatechin Gallate (EGCG) with Cystamine. The loaded drug (Pitavastatin Calcium) can be released due to cleaving of the disulfide bonds in cystamine which occurs in response to increased oxidative stress in blood vessels. Synergistic anti‐inflammatory effect and inhibition of smooth muscle cell proliferation are achieved by combination therapy of Pitavastatin calcium with EGCG. B_2_, B_3_) Results of animal experiments showing mean neointimal area and percentage of neointimal stenosis, respectively. Reproduced with permission.^[^ ^172^ ^]^ Copyright 2021, American Chemical Society.

Although drug‐eluting stents are successful in reducing the risk of restenosis compared to bare metal stents, they still have some drawbacks such as stent thrombosis due to inhibition of endothelialization and the need for prolonged antiplatelet therapy for patients after stent implantation.^[^ ^168^ ^]^ To overcome this issue, a proliferation‐induced drug release was reported by Gliesche et al.^[^ ^171^ ^]^ They developed a biodegradable poly‐l‐lactic acid (PLLA) stent where model drugs are covalently conjugated to the surface by cleavable peptide linkers (**Figure** **10**A_1_). They demonstrated that the peptide linker can be cleaved, and the model compound fluorescein can be selectively released by matrix metalloproteinase‐9 (MMP‐9) secretion. MMP‐9 is secreted in human coronary artery smooth muscle cells (HCASMC) proliferation and not proliferating human coronary artery endothelial cells (HCAEC) (Figure 10A_2_). Fluorescein release was observed only in the presence of proliferating HCASMC (Figure 10A_3_). Although these findings suggest a promising method for selective release of drugs, further studies need to be performed to prove the safety and efficacy of this method in vivo.

Another method used to achieve controlled release was the use of a pH‐responsive matrix filled with the drug. Since the angioplasty site shows inflammation which creates a slightly acidic microenvironment compared to normal tissue, Lu et al. introduced an unorthodox strategy for the treatment of cardiovascular disease. They developed a pH‐responsive coating on a stent loaded with Hydrogen sulfide donor (ACS14) as an intelligent drug release system adjusted specifically to the acidic and inflammatory environment of the intervention.^[^ ^164^ ^]^ Hydrogen sulfide is a signaling molecule that plays a vital role in maintaining cerebral vascular homeostasis, protecting, and regulating the central nervous system, promoting angiogenesis and anti‐inflammatory mediators. It can also be utilized in vascular remodeling and regulating the vascular inflammatory response. Hydrogen sulfide, however, is cytotoxic at high dosages, has a short lifetime, and cannot be directly administered. To embed the ACS14 into a pH‐responsive coating, first the surface of stainless‐steel stents was modified with dopamine. Next, the pH‐responsive coating was made layer‐by‐layer using catechol‐modified chitosan and catechol‐modified hyaluronic acid. This polymeric coating displayed increased swelling in response to the acid microenvironment of the implantation lesion. As a result, the coating was able to regulate the release of the ACS14 according to the microenvironment of the stent with a pH‐dependent release profile. In vitro studies showed that platelet adhesion and activation as well as fibrinogen adsorption and denaturation can be inhibited using this drug‐loaded coating. Proliferation of smooth muscle cells and macrophages were also inhibited, reducing the inflammatory response at the interventional site as well as promoting the formation of new blood vessels.

Coronary stent implantation can alter levels of reactive oxygen species and negatively affect the function of endothelial and smooth muscle cells. These events can ultimately cause conditions such as restenosis, thrombosis, or endothelial dysfunction in the treated artery.^[^ ^173^ ^]^ In a similar approach, a smart coating was developed on the surface of a cardiovascular stent that can release therapeutics in response to oxidative stress (at vascular lesion sites) (Figure 10B_1_).^[^ ^172^ ^]^ The coating layer was composed of a mixture of epigallocatechin gallate (EGCG) and cysteine hydrochloride, which were loaded with pitavastatin calcium. EGCG and pitavastatin calcium provided concurrent antioxidant activity and inhibition of smooth muscle cell proliferation. Specifically, smart release of drug in response to reactive oxygen species was provided via breaking of disulfide bonds of cystamine. The rate of drug release had a direct correlation with the degree of oxidative stress. In vitro observations showed that the coating possessed favorable properties such as promotion of endothelial cell proliferation and migration, in conjunction with inhibition of SMC proliferation. Moreover, animal experiments proved the biosafety of the coatings and the ability to concomitantly promote endothelialization while inhibiting neointimal hyperplasia after stent implantation (Figure 10B_2_,B_3_). The obtained results suggested that oxidative stress‐responsive coatings can be a viable and effective option for smart release of therapeutics from cardiovascular stents.

Drug eluting stents for local release of drugs at the lesion site have revolutionized the field of interventional therapies. Particularly, controlled drug release in response to conditions of the lesion microenvironment are of much interest in personalized treatments. Along these lines, enzyme‐ and pH‐sensitive polymer coatings were employed to facilitate smart‐release of therapeutics in response to diseased tissue microenvironments. Another approach to instigate on‐demand release of therapeutics is the use of externally applied signals in the form of ultrasonic or magnetic pulses. This allows for drug presentation at the ideal time and at the ideal dosage needed for a specific condition in a particular patient.^[^ ^174^ ^]^

A summary of therapeutic‐releasing biopolymeric coatings on cardiovascular implants is provided in **Table** **4**.

**Table 4:** Summary of therapeutic‐eluting cardiovascular stents

Type of implant	Type of biopolymer(s) coating	Therapeutic Type	Mode of delivery	In vivo model	Results	Ref.
Cardiovascular (stent)	Double layer coating of zein and crosslinked alginate	Rutin	Passive	–	Release profile was expanded to 21 days	[163]
Cardiovascular (stent)	Poly(l‐lactide‐*co*‐ caprolactone) (PLCL)	Combination of atorvastatin and fenofibrate	Passive	Rat subcutaneous model	Sustained release of both drugs for more than 60 days was achieved.	[162]
Cardiovascular (stent)	Chitosan and poly‐l‐lactic acid (PLLA)	A monoclonal antibody (SZ‐21), vascular endothelial growth factor (VEGF121) and rapamycin (RAPA)	Passive	Stent implantation into a left carotid artery of a rabbit	Re‐endothelialization was accelerated, and thrombosis, inflammation and in‐stent restenosis were inhibited in the multiple drug‐eluting stents after 4 weeks and 12 weeks	[165]
Cardiovascular (stent)	Chitosan and PLGA	A monoclonal antibody (SZ‐21) and docetaxel (DTX)	Passive	Porcine coronary artery model	Re‐endothelialization was promoted while neointimal hyperplasia was inhibited when a drug‐loaded hydrophobic core/hydrophilic shell particle coating stent was implanted	[166]
Cardiovascular (stent)	Poly‐l‐lysine (PLL) and hyaluronic acid–dopamine conjugate (HA–DA)	A nitric oxide donor	Passive	Porcine coronary injury model	Release profile was maintained for 5 days. Reduced inflammation, re‐endothelialisation and stronger inhibition of neointimal hyperplasia was observed in in vivo tests.	[167]
Cardiovascular (stent)	Polyglycidyl methacrylate (PGMA)	Heparin/nitric oxide donor nanoparticles	Passive	Rabbit carotid artery stent implantation	Drug‐eluted stent induced accelerated endothelial cell regeneration and kept good anticoagulant activity after 1 month of implantation	[169]
Cardiovascular (stent)	Electrospun polylactic acid (PLA) fibers	Paclitaxel and vascular endothelial growth factor (VEGF)	Passive	Canine vein pouch aneurysm model in beagles dogs	Immediate‐ and mid‐term complete aneurysm occlusion rates were improved, and earlier endothelialization was promoted in drug‐covered stent implants compared to the bare metal stent. Better lumen restenosis was also achieved using drug covered stents in vivo	[170]
Cardiovascular (stent)	Hyaluronic acid/chitosan (HA/Chi) film	Chi‐siRNA nanoplexes	Passive	–	*Ex vivo* studies show successful delivery of siRNA into the porcine artery wall and the possible inhibition of thrombosis after implantation	[168]
Cardiovascular (stent)	Poly‐l‐lactic acid (PLLA) and the MMP‐9 cleavable peptide linkers	Model compound fluorescein	Active‐ linker is cleaved in the presence of MMP‐9	–	Smooth muscle cell proliferation triggered drug release was demonstrated using this technique.	[171]
Cardiovascular (stent)	Hyaluronic acid and chitosan	Hydrogen sulfide (H_2_S) releasing aspirin derivative ACS14	Active in response to pH changes	Abdominal aorta of male Sprague–Dawley rats	Significantly smaller new tissue area was formed in implanted stents with drug‐loaded coatings into the abdominal aorta of male Sprague‐Dawley rats compared to the bare metal stents after 30 days of implantation	[164]
Cardiovascular (stent)	Epigallocatechin Gallate (EGCG) Crosslinked with cystamine (Cys)	Pitavastatin Calcium (Pi)	Active‐ in response to reactive oxygen species (ROS)	Iliac arteries of New Zealand rabbits	In vitro, no cytotoxicity was observed to endothelial cells; pitavastatin calcium was capable of promoting endothelial cells growth, while inhibiting that of SMCs. After three months of implantation in animal models significant reduction in neointimal hyperplasia was observed.	[172]

## 6 Conclusion and Future Trends

In this work we have reviewed the most recent advances in the field of drug‐eluting implants, for both hard‐ (bone) and soft‐tissues (brain, cardiovascular, and ocular). Coating of implants with drug‐loaded multifunctional biopolymers allows precise control over local release of therapeutics, which subsequently leads to enhanced implant performance and better integration within the host tissue. However, the selection of biopolymeric coating can be an intricate task. The coating must provide a surface that mimics the physicochemical properties of the host tissue to promote the integration of the implant and also provide an environment that allows for high drug loading capacity and the desired drug delivery profile over time.^[^ ^175^, ^176^ ^]^ The selection of the biopolymer is based on the difficult balance of all these properties. For example, for some bone implants, biomaterials should resist deformation and should be able to support heavy loads. However, at the same time, the same material must be flexible, to avoid cracking when exposed to tension, as well as light to enable motion.^[^ ^2^, ^177^, ^178^ ^]^ For neural microelectrode implants, the biomaterial coating or sheathing should encourage neurite outgrowth toward the surface of the electrode, while avoiding the recruitment of glial cells and fibroblasts which can lead to glial scarring, compromising activity. The microelectrodes of neural implants must also maintain electrochemical and physical stability in an adverse tissue environment to sustain the efficacy of the device for several years.^[^ ^175^, ^176^, ^179^, ^180^ ^]^ Coatings for implants for heart and coronaries must demonstrate exceptional biocompatibility with blood and enable blood flow optimization. This will prevent hemodynamic adverse responses, including thrombogenesis, which could lead to implant rejection and potentially lead to death.^[^ ^181^ ^]^ For ocular release, the biomechanical and optical properties of the implants should also guarantee they do not affect the vision or restrict oxygen permeability, ensuring biocompatibility. The implants should also have a moist surface and adequate flexibility to prevent tissue irritation.^[^ ^182^ ^]^ Another consideration is that tissue‐type can determine the nature of the stimuli that can be used to stimulate active‐release of therapeutics. Electrical pulses have been used to initiate release of therapeutics from brain implants, whereas photoradiation was used to allow on‐demand release in ocular implants. Biological changes in diseased tissue microenvironment can also be used as stimuli to initiate the release of therapeutics. For instance, enzymes and pH changes associated with infections have commonly been used as triggers to facilitate release from bone, brain, ocular, and cardiovascular implants.

The future direction of delivery from medical implants will be determined by the development of new biopolymeric entities. Given the importance of cellular penetration of therapeutics, nanogels capable of breaking down into nanoparticles are an ideal candidate for surface coating implants.^[^ ^183^, ^184^ ^]^ Targeting moieties can be added onto these nanogels to specifically target certain cell‐types within the tissue. Furthermore, theranostic nanogels (containing the therapeutic, targeting ligands, and an imaging component) can be used for simultaneous diagnosis and treatment.^[^ ^185^ ^]^ Another emerging area of research within biopolymeric coating of implants is the application of antimicrobial polypeptides in combination with antibiotics. Most bacteria can inhibit the action of antibiotics by limiting their intracellular accumulation, hence membrane‐active antibacterial polypeptides can be used as an encapsulating matrix to achieve synergistic effects.^[^ ^186^ ^]^

Biopolymers sensitive to both internal and external stimuli have been successfully used to allow on‐demand release of therapeutics. However, for successful transition of these biopolymeric coatings to clinical applications, they must comply with multiple conditions. They should be nontoxic and not act as an irritant when implanted. Second, the biopolymers degradation profile must be tuned to the specific application in order to persist in the implanted location for as long as necessary, but slowly degrade into safe subunits when no longer needed. Third, the biopolymeric coating should be easy to apply and ideally would be readily refillable for repeating administration. Fourth, the cost associated with synthesis and coating of biopolymers should be taken into consideration, as bench‐to‐bedside transition could be an exhaustive and expensive process. Last obstacle is the acceptance by industry and regulatory bodies, as often these innovations do not have a well‐established preclinical testing modality nor any specific classification to obtain their approval.

The market for therapeutic‐releasing biomedical implants is one that is growing. The advantages that this delivery route presents over more traditional therapeutic delivery modalities, such as oral tablets, make it likely that this biotechnology will continue to grow and that the number of biomedical implants on the market will increase. Some of the advantages of these implants are: patient compliance, stability of drugs within these devices, enhanced integration in the tissue, and longer lifetime. Overall, local delivery of therapeutics from medical implants is a promising approach that provides performance enhancement, leading to significant improvements in the patient's quality of life.

## Conflict of Interest

J.C. is a co‐founder and shareholder of TargTex S.A. Targeted Therapeutics for Glioblastoma Multiforme. All other authors declare no conflict of interest.
